# Case Report: α-Spectrin Mutation Associated with αLELY Polymorphism Responsible for Hereditary Pyropoikilocytosis

**DOI:** 10.3390/hematolrep14040043

**Published:** 2022-10-08

**Authors:** María Sánchez Villalobos, Eduardo Salido Fiérrez, Jorge Martínez Nieto, Mª Carmen García Garay, Asunción Beltrán Videla, Ana Belen Pérez Oliva, Miguel Blanquer Blanquer, José María Moraleda Jiménez

**Affiliations:** 1 Servicio de Hematología, Hospital Clínico Universitario Virgen de la Arrixaca, 30120 Murcia, Spain; 2 Instituto Murciano de Investigación Biosanitaria (IMIB)-Arrixaca, 30120 Murcia, Spain; 3 Servicio de Hematología, Hospital Clínico San Carlos, 28040 Madrid, Spain; 4 Centro de Investigación Biomédica en Red de Enfermedades Raras (CIBERER), 28029 Madrid, Spain

**Keywords:** congenital hemolytic anemia, red blood cell membrane, pyropoikilocytosis

## Abstract

Hereditary pyropoikilocytosis (HPP) is characterised by severe hemolytic anemia due to membrane instability. We report the case of a 13-day-old boy with neonatal jaundice and severe hemolytic anemia. A peripheral smear examination showed severe anisopoikylocytosis. DNA sequencing revealed compound double heterozygous for mutant α-spectrin SPTA1 (Arg28His) and homozygous αLELY polymorphism (low expression α-spectrin allele), compatible with diagnosis of HPP.The patient required a blood transfusion initially, but spontaneously improved after two years. Our case illustrates that, despite the presence of the allele αLELY in homozygous, the clinical phenotype is similar to cases with a mutation in SPTA1 associated with αLELY in trans.

## 1. Introduction

Hereditary pyropoikilocytosis (HPP) is a subset of hereditary elliptocytosis (HE) due to homozygous or compound heterozygous mutations in spectrin, leading to severe disruption of spectrin self-association due to homozygous or compound heterozygous mutations in α-spectrin, leading to severe disruption of α-β spectrin dimer self-association. This leads to a disruption of the horizontal junctions of the cytoskeleton of the red blood cell membrane, with loss of elasticity and fragmentation. HPP is characterised by severe hemolytic anemia with microspherocytes, poikilocytes, and an unusual thermal sensitivity of red cells because of membrane instability [1,2].

α-spectrin is the predominant component of the membrane skeleton of the red cell, and it is essential in determining the properties of the membrane. Different α-spectrin abnormalities are involved in spherocytosis type 3 (phenotype MIM, number 270970), HE type 2 (phenotype MIM number 130600) and HPP (phenotype MIM number 266140) [3].

Normally, α -spectrin synthesis is threefold greater than beta-spectrin. Heterozygote defects (HE) produce sufficient α-spectrin to balance normal β-spectrin production, and, therefore, biallelic involvement is necessary for the severe phenotypic expression in HPP. Biallelic spectrin involvement is rare, but more common is the presence of widespread polymorphisms that can modulate the phenotypic expression of the disease. The most frequent is the αLELY allele (20–30% of the population), a low-expression allele of spectrin α-spectrin gene.

This allele has carrier mutations that impair the recruitment of α-chains by β-chains, and would eventually account for the low-expression character. When αLELY polymorphism is present, and it is also present in *trans*, the propensity of the normal allele to associate with the corresponding β-chain diminishes, favouring the attachment of the elliptocytogenic α-spectrin allele. In HPP, clinical severity correlates with the amount of mutant spectrin and excess of spectrin dimer in the red cell membrane. In contrast, the αLELY component in *cis* to the alpha spectrin mutation counteracts the similar component in *trans*, and, therefore, the clinical presentation is very different. However, the clinical phenotype in the presence of the homozygous αLELY component has not been reported [2,4,5,6,7].

## 2. Case Report

**Figure 1:**
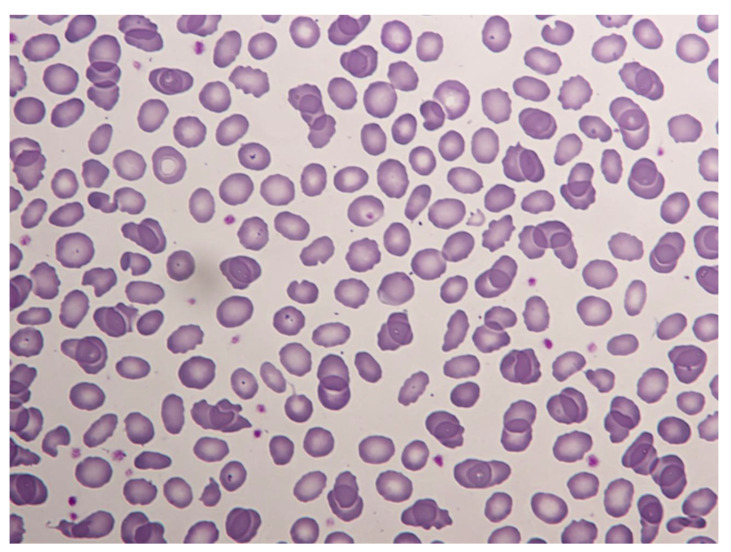
Peripheral blood smear of the patient. Severe anisopoikylocytosis with fragmented erythrocytes, spherocytes and ovalocytes. (May–Grünwald–Giemsa ×50).

**Figure 2:**
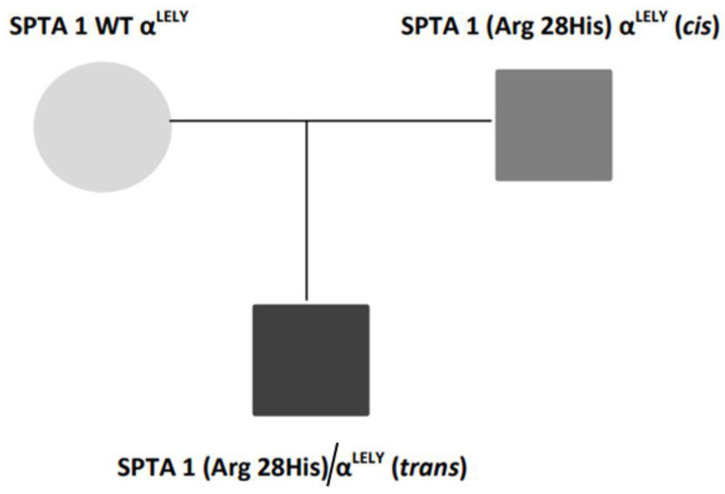
Family genetic results.

A 13-year-old Senegalese boy was referred to our hospital for neonatal jaundice and severe anemia. The blood test revealed microcytic regenerative anemia with hemoglobin levels of 60 gr/L, an increase of hemolysis markers and a negative direct antiglobulin test (Table 1). A peripheral smear examination showed severe anisopoikylocytosis with fragmented erythrocytes, spherocytes and ovalocytes consistent with hereditary membrane disorder (Figure 1). Capillary electrophoresis did not detect HBS or any other variant. The enzymatic assays of glucose-6-phosphate dehydrogenase and pyruvate kinase were normal. We performed next-generation sequencing (NGS) in a panel of 40 genes for a patient with suspected congenital hemolytic anemia. DNA sequencing revealed compound double heterozygous for mutant α-spectrin SPTA1 (Arg28His) and homozygous αLELY polymorphism (low expression α-spectrin allele), with a final diagnosis of HPP. A more complete investigation was made of his parents (Figure 2).

**Figure 3:**
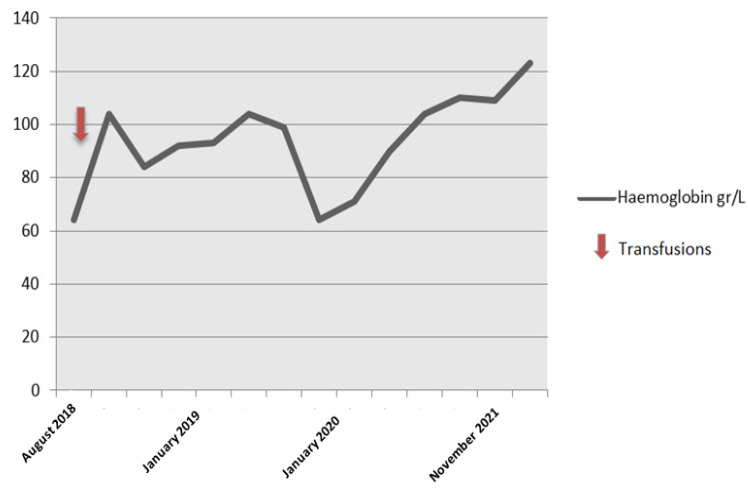
Hemoglobin levels.

The patient required blood transfusion initially but spontaneously improved after two years (hemoglobin levels 110 gr/L) (Figure 3).

## 3. Discussion

The Arg28His mutation was originally reported in a family of French descent and later in a Tunisian family. This mutation is due to a CAT-to-CGT change in codon 22, in exon 2 of the SPTA1 gene (which encodes amino acids 3 to 82 of the alpha-I domain). The defect resulted in decreased ability of the spectrin dimers to self-associate (elliptocytogenic α-spectrin chain) [8,9].

When αLELY polymorphism is present (affecting 20–30% of the population), and it is also present in *trans* (both mutations in different alpha-chain alleles), the propensity of the normal allele to associate with the corresponding beta-chain diminishes, favouring the attachment of the elliptocytogenic alpha-spectrin allele. In HPP, clinical severity correlates with the amount of mutant spectrin and excess of spectrin dimer in the red cell membrane. The phenotype improves with age, probably due to γ/β globin gene switching and a decrease of red blood cell 2,3-diphosphoglycerate [10].

HPP was originally described in 1975 as a distinct hemolytic anemia characterized by microspherocytosis, poikilocytosis, and an unusual thermal sensitivity of red cells. HPP is a subset of hereditary elliptocytosis due to homozygous or compound heterozygous mutations in spectrin, leading to severe disruption of spectrin self-association [4].

Hereditary membrane erythrocyte disorder is a rare cause of neonatal jaundice and anemia. This case highlights the importance of correlating erythrocyte morphology, the clinical course, and molecular analysis in cases of non-immune hemolytic jaundice. The finding of elliptocytogenic spectrin variants can predict the clinical course in these kind of patients. Typically, when mutation in SPTA1 is associated with αLELY in *trans*, the patients present transfusion needs in infancy, but the phenotype improves later in life [5,6,7].

Our case also illustrates that, despite the presence of the allele αLELY in homozygous, the clinical phenotype is similar to cases with *trans* presentation previously reported, and this clinical behavior supports the diagnosis of HPP rather than HE alone.

## Figures and Tables

**Table 1:** Laboratory characteristics.

Parameter	Patient (13-Day-Old)	Patient (2-Year-Old)	Mother	Father
RBC (×10^6/^L)	2.6	4.4	4.7	5.0
Hb (g/L)	60	110	125	147
MCV (fL)	70	74	79.7	83.8
MCH (pg)	24	25.1	26.5	29.7
RDW(%)	16	15	13.5	12.1
Reticulocytes (%)	5.9	6.23	1.91	1.88
Leucocytes (×10^6/^L)	6000	4830	8170	5800
Platelets (×10^6^/L)	302	278	242	222
RBC osmotic fragility	Tendency to hyporesistance/Inconclusive	---	----	---
EMA	Decrease of 11.5%	---	---	---

Hb: hemoglobin; MCV: mean corpuscular volume; MCH: mean corpuscular hemoglobin; RDW: red blood cell distribution width; RBC: red blood cells; EMA: Eosn-5-maleimide.
